# Covid-19 Effects on the Mental Workload and Quality of Work Life in Iranian Nurses

**DOI:** 10.5334/aogh.3386

**Published:** 2021-08-09

**Authors:** Kiana Nikeghbal, Bahram Kouhnavard, Ali Shabani, Zahra Zamanian

**Affiliations:** 1 Department of Oral and Maxillofacial Radiology, School of Dentistry, Shahrekord University of Medical Sciences, Shahrekord, Iran; 2 Department of Occupational Health Engineering, School of Health, Tehran University of Medical Sciences, Tehran, Iran; 3 Department of Ergonomics, School of Health, Shiraz University of Medical Sciences, Shiraz, Iran; 4 Department of Occupational Health Engineering and Ergonomics, School of Health, Shiraz University of Medical Sciences, Shiraz, Iran

## Abstract

**Introduction:**

The mental health of people working in Covid-19 wards (nurses, doctors, etc.) may be compromised due to the specific conditions of the workplace and patients. Therefore, the aim of this study was to investigate the relationship between mental burden and quality of work life in nurses in intensive care units of Covid-19 patients.

**Method:**

In this cross-sectional study, a sample of 200 people—100 nurses in care units for patients with COVID-19 (group 1) and 100 nurses in non-COVID-19 patient care units (group 2—in three university hospitals were obtained. These 200 samples were randomly extracted from the list of employees and selected. Data were collected using three questionnaires, including (1) a demographic, (2) the NASA-Task Load Index (1988) (Hart & Staveland, 1988) and (3) National Institute for Occupational Safety and Health (NIOSH) Quality of Life. Data were analyzed using SPSS-24 software and descriptive and analytical statistical methods.

**Results:**

The overall mean scores of nurses’ quality of work life were significantly different between the two groups (P < 0.05). The average score of quality of life in nurses caring for patients with COVID-19 is 92.57, more than nurses caring for patients without COVID-19, 79.43. Among the dimensions of mental workload: Performance and efficiency, with an average score of 77.32 ± 15.85, had the highest score, while discouragement and failure, with an average score of 58.04 ± 26.72, had the lowest score of mental workload. There is a significant difference between the mental load of work in the two groups (P = 0.001). There is a significant inverse relationship between total quality of work life and total mental workload (r = –14 and P = 0.01).

**Conclusion:**

In this study, it was observed that nurses caring for Covid-19 patients are in a more unfavorable situation in terms of the studied characteristics. Due to the work period, these nurses have a high workload and a low quality of work life to compensate for the mental and physical deficiencies required by a long presence in the work environment.

## Introduction

The epidemic of Covid-19 disease has affected almost all important economic, political, social, and even military aspects of the world, psychologically affecting the mental health of people at different levels of society [1]. Considering that in all countries nurses are the largest human resource in the health system [2], are in the first line of the fight against infectious diseases and Covid-19 disease, and can have a close relationship with patients, they are the first to be exposed and their physical and mental health is endangered [3].

In Iran, 80% of people working in the health care system are nurses who have undertaken 80% of the system work. High mental workload has been reported as the main source of stress in nurses and can have a negative impact on nurses’ behavior, performance, and quality of work life [4]. Workload is a multidimensional, complex concept, and is a subset of cognitive ergonomics, which is a structure for describing the extent of the physical and cognitive resources involved in performing a particular action. High-level workload is recognized as a major concern in health care that can have negative consequences on these services [5,6].

The results of research have shown that the increase in patient mortality has a significant relationship with the increase in nurses’ workload [7]. Various studies have shown that, in jobs with high mental workload–due to fatigue and improper scheduling–people’s ability to learn and work is reduced, memory and thought process are impaired, and irritability appears [8,9]. In a Radüntz study (2020) that looked at the effect of planning, strategy learning, and working memory capacity on mental workload, the workload index showed that the number of people with higher working memory capacity gradually decreased when learning a planning task strategy. However, the effect of learning on the volume of mental work in the main stage disappeared [10]. In 2017, a study by Albuquerque et al. looked at physical activity through the relationship between quality of life and memory in the elderly. The study consisted of 149 participants (both male and female, aged between 60 and 97 years) who volunteered for the study, and who were divided into two groups: Physically active seniors (who spend more than 300 min doing moderate or vigorous physical activity per week) and less active seniors. Important differences were detected between the two groups. The physically active seniors differed significantly from the less active seniors in the Mini-Mental State Examination concerning memory related items. Regarding the quality of life, the group of the physically active seniors obtained higher scores on the Quality of Life Questionnaire WHOQOL-OLD. Taken together, these results provide support for the prevailing notion that physical activity can be a prime factor in enjoying a satisfactory quality of life and in maintaining memory skills at a higher level at old age [11].

As mentioned, mental workload can have a negative impact on the quality of work life of nurses. Quality of work life can be defined from both objective and subjective perspectives. Objectively: A set of real working conditions in an organization, such as salaries and benefits, welfare facilities, health and safety, participation in decision-making, democracy, supervision, diversity and richness of jobs. Subjectively: An individual’s perception and attitude, in particular, regarding the quality of work life [12]. Although nurses represent the largest working group in hospitals and as hospital care facilitators, few studies have addressed the nature of nursing work and the quality of nurses’ working life [13]. One of these studies shows that only 1.5% of nurses are satisfied with their quality of work life [14]. The results of a study conducted in Tehran also show that two thirds of nurses are dissatisfied with the quality of their working life and are dissatisfied with most aspects of their working life [15].

Therefore, the need to pay more attention to mental health in nurses is more necessary than ever due to their valuable role in promoting and maintaining the health of clients. With the prevalence of Covid-19 disease, as well as the importance and novelty of the subject, and the fact that limited research has been conducted in this field, the purpose of this study was to investigate the relationship between mental workload and quality of work life in Covid-19 intensive care unit nurses.

## Method

### Sample Size

In this cross-sectional study, there was a mean difference of 95% confidence, 80% power, a standard deviation of 6, and the minimum acceptable difference was 2, according to the formula used (Formula 1). 200 people were selected randomly from the list of employees from three university hospitals: 100 nurses in Covid-19 patient care units (group 1) and 100 nurses in non-Covid-19 patient care units (group 2). All participants received information regarding the objectives of the research and informed consent to participate was gained. Selected candidates that did not wish to participate were replaced with the next randomly selected candidate.

N \approx 4{\sigma ^2}\left( {1 - {\rho ^2}} \right){\left( {{Z_1} - {{\alpha}/2} + {Z_{1 - \beta }}} \right)^2}

The inclusion criteria were as follows: (a) willingness to participate in the research, (b) history of providing clinical care to patients with COVID-19 for at least one month (group 1), and (c) clinical work experience for more than six months. The nurses’ unwillingness to continue cooperation with the study and complete the questionnaires was considered the exclusion criterion. The questionnaires were completed face-to-face in the hospital over ten minutes in the presence of researchers during nurses’ break.

### Tools

Data were collected using three questionnaires, including (1) a demographic, (2) the NASA-Task Load Index (1988) (Hart & Staveland, 1988), and (3) National Institute for Occupational Safety and Health (NIOSH) Quality of Life.

#### The demographic questionnaire

The demographic questionnaire inquired about age, gender, marital status, clinical work experience, ward of work, number of children, work shifts and place of work.

#### NASA-Task Load Index (NASA-TLX) questionnaire

In the present study, the NASA Workload Index was used to assess perceived mental workload. The NASA Mental Load Index (NASA-TLX) is a well-known tool for assessing mental load from an individual perspective, developed in 1988 by Hart and Steveland [16]. This tool is a multi-dimensional tool for evaluating mental workload. The NASA-TLX index includes three dimensions of needs imposed on the operator during work (physical needs, mental needs, and time needs) as well as three factors related to the result of work (personal performance, effort, level of frustration) [17]. Each dimension is rated on a 21-point Likert scale with a range of 0 to 20. Each scale is marked with a title (e.g., mental need) and a bipolar description (high-low) is given at both ends of the line. The process of assessing mental workload using the NASA-TLX model consists of three steps: The first step is to determine the weight of each of the six scales (weighting), the second step is to determine the degree of load of each of the six scales (rating), and finally, the third step is to determine the final score of the mental workload. At the end of the weighting process, a score in the range of 0 to 100 is obtained, the higher the score, the greater the mental load [18]. In this study, the total workload score is calculated using RTLX, or non-weighted, score. The use of this method is more common, because a high correlation has been observed between the weighted and non-weighted methods [19]. Studies have also shown that RTLX scores provide a better estimate of the workload experienced by the individual than the traditional weighted method. In addition, the two-stage (weighted) method prolongs the process of completing the questionnaire and makes people reluctant to participate in the study [20]. To calculate each dimension, the selected number in each dimension is multiplied by 5. In this way, the lowest score is “0” and the highest score is “100”. To calculate the final score, the values obtained from the six dimensions are averaged. The closer the score is to 100, the greater the perceived workload. The validity and reliability of this questionnaire in the nurses of special wards has also been confirmed in the country. In the study of Mohammadi et al., the reliability coefficient (Cronbach’s alpha) of this questionnaire was reported to be 0.84 in the nurses of the intensive care unit of one of the medical hospitals of Tehran [20].

#### NIOSH Quality of Worklife questionnaire

The NIOSH Quality of Worklife (QWL) module consists of 76 questions that were presented by NIOSH in 2002 in order to sort out work life issues such as working hours, workload, work uniformity, job security, job satisfaction, job stress, and health. The reliability and validity of this questionnaire has been confirmed by the staff of Shiraz University of Medical Sciences: In the study of Choobineh et al., the reliability coefficient (Cronbach’s alpha) of this questionnaire was reported to be 77%. One of the purposes of this questionnaire is to determine the relationship between job-organizational characteristics and health of individuals. The questions are scored in the form of Likert scale (two, four, and multiple choice) and open-ended questions. A lower score indicates a worse condition and a higher score indicates a higher quality of life in individuals [21,22]. The QWL score is based from 0-100: 0-33 indicates poor quality of working life; 33-66 indicates the average quality of working life; and the score of 66-100 indicates a high quality of working life. The QWL score includes the total scores from the dimensions of: Job satisfaction, job security, job uniformity, job stress, and workload. The higher the mean score of job satisfaction and job security, the better the quality of work life. The lower the average score of job uniformity, job stress, and workload means a higher job uniformity, job stress, and workload, and the worse the quality of work life of individuals [23,24]. Data were analyzed using SPSS-24 software and descriptive and analytical statistical methods.

## Results

### Sample Characteristics

In the present study, 100 nurses cared for patients with COVID-19 and 100 nurses cared for patients without COVID-19. Frequency of income, gender, marital status, education, turnover, age, work experience, and number of hours worked overtime are given, by nurse’s place of work, in ***Table 1***.

**Table 1:** Frequency of demographic characteristics by nursing workplace.


VARIABLES		NURSE’S PLACE OF WORK	

		PATIENTS WITH COVID-19 FREQUENCY	PATIENTS WITHOUT COVID-19 FREQUENCY

Income (million toman)	3–5	30	28

	5–7	62	65

	7–9	8	7

Gender	Female	37	43

	Male	63	57

Marital status	Married	72	69

	Single	28	31

Education	Diploma & Bachelor’s	85	84

	Master & Upper	15	16

Working shift	Non- Rotational	14	14

	Rotational	86	86


Some variables are given as averages and standard deviations related to the demographic characteristics displayed by nurse’s workplace in ***Table 2***.

**Table 2:** Mean and standard deviation related to demographic characteristics by nursing location.


VARIABLES	NURSE’S PLACE OF WORK MEAN (± μ)	

	PATIENTS WITHOUT COVID-19 FREQUENCY	PATIENTS WITH COVID-19 FREQUENCY

Age	30.08(±2.04)	30.19(±2.04)

Work experience	4.91(±2.22)	4.92(±2.21)

Number of working hours	138.7(±43.10)	236.5(±44.61)

Overtime hours	102.20(±30.60)	109.30(±31.82)


***Table 3*** shows the relationship between quality of work life and demographic variables by nurse’s workplace.

**Table 3:** Relationship between quality of work life (mean) and demographic variables by nurse workplace.


VARIABLES		NURSE’S PLACE OF WORK			

		PATIENTS WITHOUT COVID-19		PATIENTS WITH COVID-19	

		MEAN	P_VAL_	MEAN	P_VAL_

Employment Status	Contractor	60.06	0.21	86.55	0.35
	
	Contractual	60.30		86.11	
	
	Contractual or Formal	63.29		85.11	

Income (million toman)	3–5	50.02	0.041	63.48	0.042
	
	5–7	58.07		63.11	
	
	7–9	63.66		65.22	
	
	>9	63.06		66.98	

Gender	Female	50.27	0.089	53.20	0.04
	
	Male	58.97		63.00	

Marital status	Married	70.11	0.036	58.30	0.001
	
	Single	61.10		50.06	

Education	Diploma & Bachelor’s	63.33	0.032	53.63	0.143
	
	Master & Upper	68.17		60.29	


In ***Table 4***, considering that the results of the QWL questionnaire and demographic variables such as weight, height, work experience, working hours, and overtime hours are all quantitative data, the use of Pearson statistical tests demonstrates a significant level of quality of life. Work with demographic characteristics is presented separately for nurse’s workplaces.

**Table 4:** Significant level of quality of work life with demographic characteristics by nurse workplace.


VARIABLES	NURSES CARING FOR PATIENTS WITH COVID-19 (P_VAL_)	NURSES CARING FOR PATIENTS WITHOUT COVID-19 (P_VAL_)

Weight	0.110	0.350

Height	0.135	0.533

Work experience	0.192	0.113

Working hours	0.554	0.754

Overtime hours	0.128	0.233


According to ***Table 4***, none of the quantitative demographic characteristics such as weight, height, work experience, working hours, or overtime had a statistically significant relationship with quality of working life (p > 0.05). Considering that a comparison of the mean quality of working life between the two groups of nurses is required, ***Table 5*** shows the results of comparing the average quality of working life between the two groups using paired-sample t-test.

**Table 5:** Comparison of the average quality of working life between the two groups of nurses.


VARIABLES	NURSES CARING FOR PATIENTS WITH COVID-19 MEAN ± μ	NURSES CARING FOR PATIENTS WITHOUT COVID-19 MEAN ± μ	P_VAL_

Overall quality of work life	49.43 ± 9.47	62.57 ± 15.93	0.001

Fair and adequate payment	0.327 ± 0.23	2.234 ± 0.31	0.001

Safe and hygienic environment	0.213 ± 0.3	4.48 ± 1.64	0.001

Provide opportunities for continuous growth and security	0.73 ± 0.1	8.5 ± 1	0.001

Legalism in the job organization	11.54 ± 1.9	11.12 ± 1.23	0.54

Social dependence on working life	0.11 ± 0.25	2.14 ± 1.05	0.001

The general atmosphere of work life	6.36 ± 1.65	11.62 ± 2	0.001

Social integration and cohesion	0.26 ± 0.31	9.8 ± 1.43	0.001

Capability development	0.48 ± 0.83	8.4 ± 1.86	0.23


As shown in ***Table 5***, the independent paired t-test showed that the overall mean scores of nurse’s quality of work life were significantly different between the two groups (P < 0.05). The average score of quality of life in nurses caring for patients with COVID-19 is 92.57, more than nurses caring for patients without COVID-19, 79.43. Findings on assessing the mental workload based on the NASA-TLX questionnaire are shown in ***Table 6***.

**Table 6:** Descriptive characteristics of the dimensions of nurses the mental work load.


VARIABLES	MEAN ± μ	MIN/MAX

Mental stress	75.35 ± 17.30	25/100

Physical pressure	65.40 ± 21.86	5/100

Time pressure	72.76 ± 18.05	10/100

Performance	77.32 ± 15.85	10/100

Attempt and effort	74.50 ± 17.97	10/100

Discouragement and failure	58.04 ± 26.72	5/100

Mental burden of work	70.44 ± 12.88	39/100


Among the dimensions of mental workload, performance and efficiency—with an average score of 77.32 ± 15.85—had the highest score with discouragement and failure last at an average score of 58.04 ± 26.72. A comparison of the workload of the two groups is given in ***Table 7***. According to this table, there is a significant difference between the mental load of work in the two groups (P = 0.001).

**Table 7:** A comparison of the mental workload of the two groups.


MENTAL WORKLOAD	MEAN ± μ	P_VAL_

Nurses caring for patients with COVID-19	93.68 ± 13.2	0.001

Nurses caring for patients without COVID-19	70.97 ± 12.57	


The correlation between the quality of work life and the mental burden of work is -0.14. There is a significant inverse relationship between total quality of work life and total mental workload (r = –14 and P = 0.01). In both groups of nurses, there is a significant inverse relationship between quality of work life and mental workload (***Table 8***).

**Table 8:** Investigating the relationship between quality of work life and mental workload in two groups.


VARIABLES		THE CORRELATION COEFFICIENT PEARSON	P_VAL_

Nurses caring for patients with COVID-19	Quality of working life	–0.16	0.001

	Mental workload		

Nurses caring for patients without COVID-19	Quality of working life	–0.25	0.01

	Mental workload		


## Discussion

The results show that most of the demographic variables in the two conditions of care for the above patient groups are not significantly different. The average score of quality of life in Covid-19 care nurses is higher than non-Covid-19 care nurses. Both groups had moderate quality of work life. In the study of Dehghan Nairi et al., the quality of work life of nurses in the middle to lower level was 61.4%, and only 3.6% of them felt satisfied with their job [25]. This is consistent with the present study. In the study of Saber and his colleagues, the quality of work life was reported to be moderate [26]. The results of current study are somewhat inconsistent with the results of research by Hesam et al [27]. In contrast, al-Maliki’s results in Saudi Arabia showed that most of the nurses studied had a low quality of working life [28]. The reason for the difference in the findings of the aforementioned studies with the present study is likely the difference in the statistical population, the tools used, and the method of scoring.

Another important result in the current study is the quality of working life in the nurses studied. In this study, it was reported that the quality of working life in nurses who care for a patient in non-Covid-19 units is higher than nurses caring for a Covid-19 patient. Accordingly, some indicators of quality of working life, such as providing opportunities for growth and continuous security, social dependence of work life, and the index of general working life space, are higher in nurses who do not care for patients with Covid-19. The reason for this difference is the type of work environment and the desired job. In nurses, caring for a Covid-19 patient can cause many hours, even days, away from the nurses’ emergency room.

In the study of Dargahi et al., the relationship between monthly income level and quality of working life is significant; the quality of work life increases with increasing salaries and benefits [29]. It seems that in his study, considering that about 85% of the nurses studied have a bachelor’s degree and a high income, there’s satisfaction. A study in Canada also found that increasing income levels increases the quality of working life [30]. But in the present study, only in nurses caring for Covid-19 patients, is the relationship between quality of work life index, such as fair and adequate pay and monthly income, significant.

Additionally, in the study by Dargahi et al., there was no significant relationship between marriage and quality of life, which is not consistent with the results of the present study. But in Khaghanizadeh’s and Fallahee’s studies, this relationship was significant [29,31,32]. In the present study, in both groups of nurses studied, married people have a better quality of work life. A possible cause of this discrepancy in the results is the non-distribution of married and single individuals in the sample size.

In the current study, the mean working hours and the overtime for nurses caring for Covid-19 patients were significantly higher than nurses caring for non-Covid-19 patients. This result is consistent with the findings of other studies [32,33,34]. The likely reason for this similarity in the results is that nurses caring for Covid-19 patients spend a lot of time in the workplace and are subjected to high work pressures—this factor can be a reason for lower quality of working life.

None of the demographic characteristics, such as employment status, gender in occupational satellite nurses, or education level in both groups were statistically significantly related to quality of working life. These findings indicate that employment status, gender in nurses caring for Covid-19 patients, and level of education cannot be used as a predictor variable for quality of working life. This suggests that these factors are not important in making a difference in the quality of work life. Also, in the study of Habibi et al., which was conducted with the aim of determining the factors affecting the quality of work life of nurses employed by magnesium companies in 2013, no significant difference was found between gender, age group, education level, work experience, income and quality of work life [35]. Different factors can affect the quality of work life and depending on the society under study; working conditions and culture can be affected by different demographic characteristics.

The results of the present study show that the average mental workload in the two groups of nurses is high. In each job, there is a certain degree and average of work-related stress psychologically, and the behavior, performance, and, consequently, productivity of people in the workplace will be affected in some way by the mental workload. In this study, it was found that nurses caring for a Covid-19 positive patient and those caring for a non-Covid-19 patient have a certain degree of mental workload, so that the assessment of mental workload shows that both of these nurse groups measure a high average mental workload. This high mental workload may be due to the high time sensitivity of work tasks in both groups of nurses.

The mental burden of nurses working in these job groups is high. Various factors, such as constant and uniform work, duration of work, job requirements (concentration, accuracy, and effort), fatigue due to physical stress, age, work experience, environmental factors (sound, vibration, etc.), working with equipment, individual feedback on work/interpersonal work interaction, overtime, and ergonomic working conditions are involved in creating and increasing the mental and psychological burden of work [36]; therefore, it can be said that these factors are among those that play a role in increasing the mental workload of nurses. Because these employees work in a consistent and stable work environment—due to the nature of the job and working conditions—they have longer working hours, which in turn causes physical and mental fatigue. Numerous studies have shown that in uniform jobs, despite low job needs, the amount of mental workload is high and increases employee fatigue [37,38]. Many studies show that jobs with a high mental load, due to fatigue and improper scheduling, reduce efficiency, memory and learning; damage the thought process; and increase irritability [39,40,41]. Additionally, tired people are more likely to engage in risky behaviors, such as taking shortcuts to accomplish their tasks [34,35,36,37].

One of the limitations of the present study is the use of a data collection questionnaire which, despite explanations given about the objectives of the research before the questionnaires were presented, could affect the way they responded dependent upon the respondents’ moods. Due to the lack of free time, high workload was initially predicted as the main hurdle to nurse participation in this study, therefore study researchers attempted to maintain continuous presence in different units and during different shifts. Researcher follow-up attempted to increase the participation in this study. Additional complications were that the questions had to be completed in the workplace where nurses had little focus on completing the questionnaires. Some nurses were reluctant to complete the questionnaires because they had not received adequate feedback from the results of previous research.

## Conclusion

As the results showed, the overall mental workload and quality of work life for nurses is not in favorable. The type of nursing service provided, including Covid-19 patient care, alters the measure of these characteristics. As in this study, it is well observed that nurses caring for patients with novel coronavirus disease (Covid-19) show higher unfavorable values. Due to the work period, these nurses have a high workload and a low quality of work life to compensate for the mental and physical deficiencies required by a long presence in the work environment.
